# Investigation of True High Frequency Electrical Substrates of fMRI-Based Resting State Networks Using Parallel Independent Component Analysis of Simultaneous EEG/fMRI Data

**DOI:** 10.3389/fninf.2017.00074

**Published:** 2017-12-22

**Authors:** Sreenath P. Kyathanahally, Yun Wang, Vince D. Calhoun, Gopikrishna Deshpande

**Affiliations:** ^1^Department of Electrical and Computer Engineering, AU MRI Research Center, Auburn University, Auburn, AL, United States; ^2^Department of Clinical Research/AMSM, University of Bern, Bern, Switzerland; ^3^Department of Psychiatry, Columbia University, New York, NY, United States; ^4^Mind Research Network and Lovelace Biomedical, Environmental Research Institute, Albuquerque, NM, United States; ^5^Department of Electrical and Computer Engineering, University of New Mexico, Albuquerque, NM, United States; ^6^Department of Psychology, Auburn University, Auburn, AL, United States; ^7^Alabama Advanced Imaging Consortium, Auburn University and University of Alabama Birmingham, Birmingham, AL, United States

**Keywords:** resting state brain networks, default mode network, primary visual cortex, simultaneous EEG-fMRI, parallel independent component analysis, neurophysiological basis of DMN

## Abstract

Previous work using simultaneously acquired electroencephalography (EEG) and functional magnetic resonance imaging (fMRI) data has shown that the slow temporal dynamics of resting state brain networks (RSNs), e.g., default mode network (DMN), visual network (VN), obtained from fMRI are correlated with *smoothed and down sampled versions* of various EEG features such as microstates and band-limited power envelopes. Therefore, even though the down sampled and smoothed envelope of EEG gamma band power is correlated with fMRI fluctuations in the RSNs, it does not mean that the electrical substrates of the RSNs fluctuate with periods <100 ms. Based on the scale free properties of EEG microstates and their correlation with resting state fMRI fluctuations in the RSNs, researchers have speculated that truly high frequency electrical substrates may exist for the RSNs, which would make resting fluctuations obtained from fMRI more meaningful to typically occurring fast neuronal processes in the sub-100 ms time scale. In this study, we test this critical hypothesis using an integrated framework involving simultaneous EEG/fMRI acquisition, fast fMRI sampling (*TR* = 200 ms) using multiband EPI (MB EPI), and EEG/fMRI fusion using parallel independent component analysis (pICA) *which does not require the down sampling of EEG to fMRI temporal resolution*. Our results demonstrate that with faster sampling, high frequency electrical substrates (fluctuating with periods <100 ms time scale) of the RSNs can be observed. This provides a sounder neurophysiological basis for the RSNs.

## Introduction

Functional magnetic resonance imaging (fMRI) reveals spontaneous low-frequency (<0.1–0.15 Hz) fluctuations of a large number of anatomically separate brain areas that are temporally correlated and functionally linked to each other at rest and these brain areas are called “resting state networks” (RSNs) (Biswal et al., 1995; Greicius et al., 2003; Fox and Raichle, 2007). It has been observed that task related response accounts for <5% of cerebral metabolism, whereas most of cerebral metabolism corresponds to resting state activity (Raichle and Mintun, 2006). Multiple studies in the previous decade have shown that the alterations in resting state brain connectivity can be associated with neurological or psychiatric diseases (Fornito and Bullmore, 2010). Therefore, RSNs have become a very popular method for assessing brain function in patient populations because it is possible to perform fMRI without stimulation which is helpful for such subjects who can have difficulties while performing tasks (Auer, 2008).

Even though blood oxygenation level dependent (BOLD) fMRI has been used to detect spatially consistent RSNs across subjects, it is still unclear when using that modality alone, if the correlated resting state activity is purely neuronal in origin or is due to low frequency physiological or scanner related artifacts (Lund, 2001; Maldjian, 2001) or vascular structure (Vigneau-Roy et al., 2014). BOLD fMRI is an indirect measure of neural activity (Logothetis, 2008) and it is also unclear whether correlated resting state activity observed from very slow fluctuations with periods of the order of 10 s of seconds are related to neuronal dynamics which occur in the sub-100 ms time scale.

Contrary to fMRI, electroencephalogram (EEG) is a direct measure of electrical activity in the brain measured at its scalp. It is the sum of the synchronous activity of neurons in the area below the electrode on the scalp (Arieli et al., 1996; Tsodyks et al., 1999). There has been some evidence showing that spontaneous resting state EEG activity fluctuates coherently and they are macroscopically organized across the brain (Laufs, 2008). Though EEG has very high temporal resolution for measuring resting state neuronal activity, its spatial resolution is very poor and hence limits our ability to make physiologically meaningful inferences about the neural basis of RSNs observed from fMRI.

In order to noninvasively understand the neural and electrical basis of RSNs observed from fMRI in humans, we need to combine EEG and fMRI signals such that we get both high spatial and temporal resolution of the underlying neuronal activity (Horwitz and Poeppel, 2002; Debener et al., 2006). There have been several studies related to simultaneous acquisition of EEG and fMRI signals which show that the EEG power envelope at specific frequency bands correlates with fMRI signals in many different RSNs (Goldman et al., 2002; Laufs et al., 2003; Moosmann et al., 2003). However, EEG is made up of a wide frequency spectrum (Varela et al., 2001; Buzsaki and Draguhn, 2004), and hence the correlation between neuronal activities only at specific frequencies and the BOLD RSNs limits possible interpretation (Laufs et al., 2006). It has been shown that the time course of RSNs can be associated with EEG power envelopes at more than one frequency band (Mantini et al., 2007). Similar studies have been carried out with magnetoencephalography (MEG) (de Pasquale et al., 2010; Brookes et al., 2011). There are two fundamental issues regarding the approaches described above. First, the EEG/MEG power envelope corresponding to various EEG/MEG frequency bands still represents a low frequency amplitude modulated signal, even though the frequency bands themselves can be of high frequency. Second, in order to match the temporal resolution of EEG/MEG to that of fMRI, these studies downsampled EEG/MEG data/power to that of fMRI temporal resolution. These two factors make it impossible to assess whether the correlations between EEG/MEG power envelope and BOLD RSNs, such as the default mode network (DMN), have a neural basis in millisecond-scale fast neuronal dynamics. However, they do point to the fact that RSNs have an electrical basis and cannot be purely based on BOLD-based physiological artifacts (Bridwell et al., 2013) or vascular structure (Vigneau-Roy et al., 2014).

Recently, EEG microstates have been shown to be scale-free and have been proposed as potential electrophysiological substrates of spontaneous BOLD activity (Britz et al., 2010; Musso et al., 2010). There is speculation that this points to the existence of truly high frequency electrical substrates of RSNs, which would make resting fluctuations obtained from fMRI more meaningful with respect to typically occurring fast neuronal processes in the sub-100 ms time scale. However, the idea that RSNs have a neuronal basis related to truly high frequency electrical activity (as opposed to downsampled and smoothed power envelopes) has only been explored very recently. For example, Lewis et al. showed that fast fMRI during a visual stimulation paradigm can detect oscillatory neural activity in humans (Lewis et al., 2016). Therefore, in this study, we tested this critical hypothesis for both DMN and VN, which are important commonly found RSNs (Greicius et al., 2003; Fox and Raichle, 2007). We used parallel independent component analysis (pICA) of simultaneously acquired EEG-fMRI data during resting state for fusing data from both modalities *without sacrificing the native resolutions of either modality* (Eichele et al., 2008).

In addition, we acquired whole brain fMRI data with TRs as short as 200 ms using multiband EPI sequence (Feinberg et al., 2010) (MB-EPI) to test the hypothesis that faster sampling would enable us to better detect the true high frequency electrical substrates of RSNs. ICA is one of the multivariate methods used to identify RSNs and does not require any *a priori* seed region (Calhoun et al., 2001; Beckmann et al., 2005). Here we applied ICA separately to EEG and fMRI data, to get statistically independent time courses (tICA) and statistically independent spatial maps (sICA), respectively. pICA is a second level analysis (Calhoun and Adali, 2009; Calhoun and Allen, 2013) which uses first level results to recover spatial maps from fMRI and time courses from EEG and match these components across the modalities to achieve multimodal integration for simultaneous resting state data. In contrast to Liu et al. (Liu and Calhoun, 2007; Liu et al., 2009) where they used a constraint to maximize the correlation between the mixing matrices of two modalities in pICA, here we used the same pICA algorithm and constraints to maximize the correlation but instead of running pICA algorithm once, we ran multiple pICAs to see which combination of EEG component with the fMRI-DMN or fMRI-VN component gave the highest correlation between the mixing matrices of two modalities. We further elaborate on this aspect in the following section.

In this study, we hypothesize that resting state fluctuations in fMRI may be associated with not only low frequency, but also high frequency electrical dynamics. To test this hypothesis, we acquired simultaneous EEG and fMRI data with faster fMRI sampling and combined them using pICA which enabled us to evaluate relationships between resting state network maps obtained from the fMRI and electrical time courses obtained from the EEG.

## Materials and methods

### Subjects, tasks, and ethical approval

Six adult subjects with no history of neurological or cardiological disorders participated in this simultaneous EEG-fMRI resting state study. The subjects were instructed to lie supine, stay awake with eyes open and not to think of anything in particular. Cushions were placed inside the coil to absorb the pressure from the EEG electrodes on the head and to restrict head movement in the coil. Subjects were also provided with earplugs to avoid any harmful effect from MRI acoustic noise. The entire study for each subject contained a single session of resting state simultaneous EEG-fMRI data acquisition using both traditional EPI sequence as well as the multiband EPI sequence. We optimized the MB-EPI sequences (by reducing the flip angle) such that its SAR (specific absorption rate) was equivalent to that obtained by the regular EPI sequence (which Brain Products, the manufacturer of the EEG system, has approved for simultaneous EEG/fMRI acquisition) so as to ensure safe acquisition of EEG/fMRI. The safety of acquiring simultaneous EEG and MB-EPI data was first tested using phantoms and then author GD self-tested the acquisition procedure multiple times on himself. The phantom testing was empirical just to make sure that it is safe for the PI to self-test on himself. No temperature measurements were performed. This was necessary because it is not customary to acquire MB-EPI data simultaneously with EEG and at the time of the experiment, this was the first study to do so according to our knowledge. However, at the time that this report was being peer-reviewed, we learned that others had performed simultaneous multiband fMRI and EEG, albeit with different equipment (Lewis et al., 2016; Foged et al., 2017). In fact Foged et al showed, using temperature measurements, that it is safe to combine high density EEG with fast fMRI techniques such as multiband EPI (Foged et al., 2017). Subsequent to our in-house testing, the procedure was approved by Auburn University's Institutional Review Board (IRB) as well as the MR safety committee. This study was carried out in accordance with the recommendations of Auburn University's IRB with written informed consent from all subjects. All subjects gave written informed consent in accordance with the Declaration of Helsinki.

### fMRI data acquisition

Resting state fMRI data were collected on a 3T Siemens Verio scanner using

1. single-shot gradient-recalled EPI sequence with 29 ms TE, 1,000 ms TR, 90°Flip angle, 64 × 64 × 16 acquisition matrix and voxel size of 3.5 × 3.5 × 6.75 mm for six healthy subjects,
2. MB-EPI sequence (obtained from the University of Minnesota) with multiband factor of 8 (MB8) (Feinberg et al., 2010) with 40 ms TE, 200 ms TR, 50°Flip angle, 64 × 64 × 16 acquisition matrix and voxel size of 3.5 × 3.5 × 6.3 mm for six healthy subjects. Split slice GRAPPA (also referred to as leak block) was utilized in order to minimize spurious correlations that may appear between slices acquired simultaneously. In-plane acceleration was not used, in part to preserve SNR lost by simultaneous multi-slice acquisition. However, we used thicker slices in order to minimize signal dropout and spatial distortion.

All the fMRI data was acquired using a standard Siemens receive–only 12-channel matrix head coil. The different TEs, flip angles and slightly different voxel sizes were necessitated because we wanted to optimize the sequence for minimum TR and whole brain coverage.

### EEG data acquisition

For simultaneous EEG-fMRI acquisition, we used MR-compatible 64 channel EEG amplifiers (Brain Products, GmBH, Germany), MR-compatible EEG cap (BrainCap MR, Falk Minow Services, Herrsching–Breitbrunn, Germany) with 63 10–20 system distributed scalp electrodes and ECG electrode. We collected 10,000 data points per TR by synchronizing the EEG data acquisition clock to the MRI scanner clock using Brain Products' SyncBox. EEG data were then digitized with a sampling frequency of 5 kHz, 0.5 μV resolution, with reference to FCz and within a DC-250 Hz frequency range. For all the EEG recordings, impedance at electrodes was <20 kΩ.

### fMRI data pre-processing

The functional MRI data obtained from each subject was realigned by taking the first image as the reference for all other scans for motion correction. The images were then resliced and spatially normalized to EPI MNI template and spatially smoothed with a Gaussian filter with 6 mm FWHM using the SPM 12 toolbox (Friston et al., 1995). The fMRI signal time courses at each voxel were then detrended followed by removal of white matter and cerebrospinal fluid (CSF) signal using Data Processing Assistant for Resting-State fMRI (Chao-Gan and Yu-Feng, 2010) which is based on SPM and the Resting-State fMRI Data Analysis Toolkit (Song et al., 2011).

### EEG data pre-processing

The Brain Vision Analyzer 2.0 software (Brain Products) was used to perform preprocessing of simultaneously acquired EEG data to reduce MRI gradient artifact, cardioballistic artifact, and ocular artifact arising from the simultaneous EEG–fMRI environment as detailed below.

- For reducing MRI scanning artifacts, we used an artifact template created by averaged artifact subtraction (AAS) method (Allen et al., 2000) in which the EEG data was segmented and averaged according to the onset of each volume within a sliding window consisting of 41 consecutive volumes, and subtracted from the raw EEG data.
- For reducing cardioballistic artifacts, we used an artifact template created by moving template subtraction approach (Allen et al., 1998) in which R peaks were detected in the low-pass-filtered ECG signal and used to construct a delayed average artifact template over 21 consecutive heartbeat events in a sliding-window approach, which was subtracted from the original EEG signal.
- The Ocular artifact was removed by applying ICA (Lee et al., 1999; Delorme and Makeig, 2004). ICA was also used to remove any residual artifacts after steps (i) and (ii).

**Figure 1:**
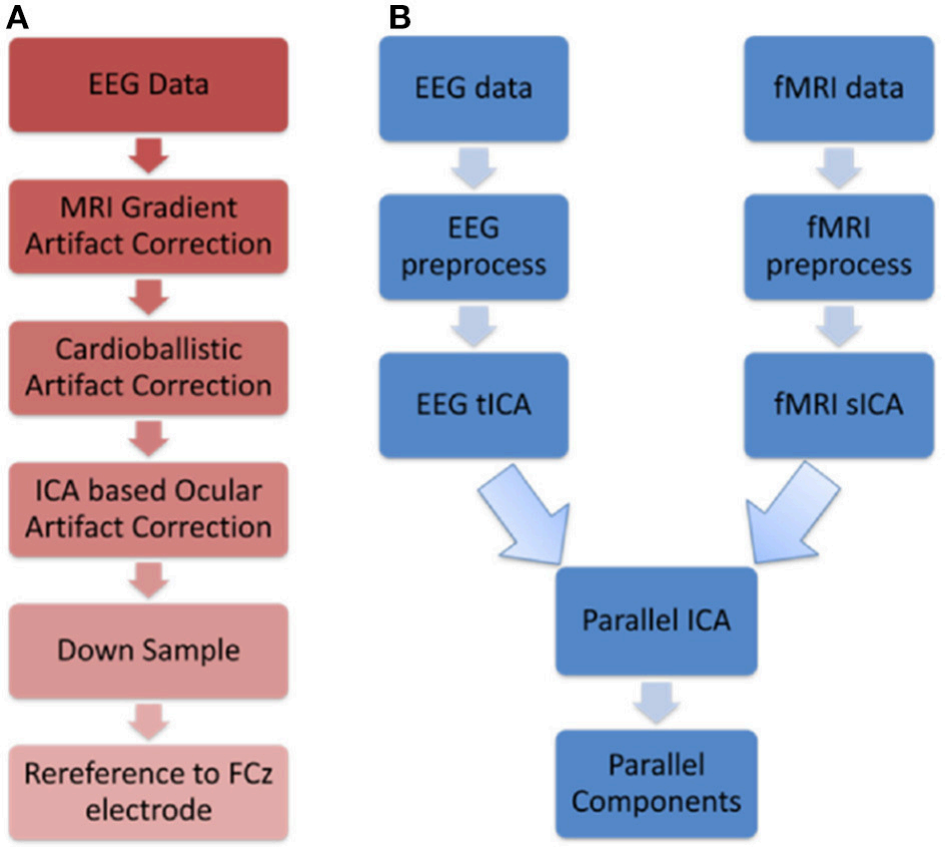
Schematics illustrating **(A)** EEG preprocessing steps before performing the first level EEG analysis using temporal ICA **(B)** Parallel ICA: The first level analysis results consisting of 20 spatial and 20 temporal components that were derived from spatial and temporal ICA, respectively, were given as input to parallel ICA.

The resulting EEG data from the above three steps were then downsampled to 250 Hz, and then re-referenced to FCz. Figure 1A shows the flowchart illustrating the EEG preprocessing steps.

### Group spatial ICA of fMRI data (sICA)

We performed group ICA on fMRI data (Calhoun and Adali, 2012) using the GIFT toolbox (Calhoun et al., 2001, 2008). The fMRI signals [*x*_1_ (*v*), *x*_2_ (*v*), …, *x*_*N*_ (*v*)], where *v* represents voxels, were assumed to be a linear mixture of statistically independent hemodynamic source locations [*s*_1_ (*v*), *s*_2_ (*v*), …, *s*_*N*_ (*v*)] such that at a given voxel, it contains a weighted mixture of the sources, *a*_*j*1_*s*_1_ (*v*) + *a*_*j*2_ *s*_2_ (*v*) + … + *a*_*jN*_*s*_*N*_ (*v*), each of which fluctuates according to its weighted hemodynamic time course, for all *j*. The weights were multiplied by each source's hemodynamic time course. The observed fMRI signal is given by, *x*_*j*_ (*v*) = *a*_*j*1_*s*_1_ (*v*) + *a*_*j*2_ *s*_2_ (*v*) + … + *a*_*jN*_*s*_*N*_ (*v*) where $x={\left[x_{1}\left(v\right),\;x_{2}\left(v\right),\;\ldots ,\;x_{N}\left(v\right)\right]}^{T},$ $s={\left[s_{1}\left(v\right),\;s_{2}\left(v\right),\;\ldots ,\;s_{N}\left(v\right)\right]}^{T}$ and *A* the mixing matrix with *a*_*ij*_ elements. We assume that the number of discrete time points acquired with the scanner is very large compared to actual sources in the brain. The fMRI data was pre-whitened to remove any correlations in the data and reduced via principal component analysis (PCA) by removing non-significant components and retaining only the principal components which contain the major proportion of variance. The principal components were then concatenated across subjects to form group data. The infomax ICA algorithm (Bell and Sejnowski, 1995) was then applied to the group data to get the independent components. 20 group components were generated as in Rosazza et al. (2012) and individual subject components were computed using back reconstruction (Erhardt et al., 2011) and the components were scaled to Z-scores to remove the arbitrary units of spatial maps and time courses during the back-reconstruction step. Finally, the mean, standard deviation and t-maps were calculated for the group data. Please note that the above steps are done automatically in the GIFT toolbox.

### Group temporal ICA of EEG data (tICA)

The EEG data was analyzed with group temporal ICA using the EEGIFT toolbox and 20 components were generated as in Eichele et al. (2011). The EEG signal [*x*_1_ (*t*), *x*_2_ (*t*), …, *x*_*N*_ (*t*)], where *t* represents time, was assumed to be a linear mixture of statistically independent non-Gaussian source time series [*s*_1_ (*t*), *s*_2_ (*t*), …, *s*_*N*_ (*t*)] such that at a given time point, it contains the weighted mixture of the sources [*a*_*j*1_*s*_1_ (*t*) + *a*_*j*2_ *s*_2_ (*t*) + … + *a*_*jN*_*s*_*N*_ (*t*)], for all *j* (Ullsperger and Debener, 2010). The observed EEG signal is given by *x*_*j*_ (*t*) = *a*_*j*1_*s*_1_ (*t*) + *a*_*j*2_ *s*_2_ (*t*) + … + *a*_*jN*_*s*_*N*_ (*t*) where $x={\left[x_{1}\left(t\right),\;x_{2}\left(t\right),\;\ldots ,\;x_{N}\left(t\right)\right]}^{T},$ $s={\left[s_{1}\left(t\right),\;s_{2}\left(t\right),\;\ldots ,\;s_{N}\left(t\right)\right]}^{T}$ and *A* the mixing matrix with *a*_*ij*_ elements. The preprocessed EEG data was pre-whitened to remove any correlations in the data and reduced via PCA by removing non-significant components and retaining only the principal components which contain the major proportion of variance. The principal components were then concatenated across subjects to form group data. The ICA was then applied to the group data to obtain the independent source time-series/components.

### Parallel independent component analysis (pICA)

Parallel independent component analysis (pICA) is a second level analysis procedure which combines spatially independent sources obtained from fMRI and temporally independent sources obtained from EEG such that the correlation between the fMRI and EEG mixing matrices is maximized. When ICA is separately applied to EEG and fMRI we assume the acquired signals to be, *X*_*EEG*_ = *A*_*EEG*_ *S*_*EEG*_ and *X*_*FMRI*_ = *A*_*FMRI*_ *S*_*FMRI*_ respectively, where *A*_*EEG*_ and *A*_*FMRI*_ are the mixing matrices for EEG and fMRI respectively and S_EEG_ and S_FMRI_ are statistically independent non-Gaussian Source time series and spatial maps, respectively. In order to determine the unmixed components *U*_*EEG*_ and *U*_*FMRI*_, we have to find *W*_*EEG*_ and *W*_*FMRI*_ such that it approximates inverse of *A*_*EEG*_ and *A*_*FMRI*_

(1) $$U_{EEG}=W_{EEG}X_{EEG}$$

(2) $$U_{FMRI}=W_{FMRI}X_{FMRI}$$

First, the dimensionality of the observed data *X*_*EEG*_ and *X*_*FMRI*_ were reduced using the Principal component analysis to get new data *Y*_*EEG*_ and *Y*_*FMRI*_ that is of the same dimension as of component matrix (*S*). Then the INFOMAX algorithm (Bell and Sejnowski, 1995), which employs gradient ascent learning rules was used to maximize the independence for the two modalities, and in determining the relationship between them. The maximization of mutual entropy (H) was used to derive the independence (as shown in equations below), and the relationship between them was determined by maximizing the squared correlation between *A* matrices.

(3) $$\begin{array}{l} \;max:\;H(Y_{EEG}),H(Y_{FMRI})\\ subject\;to:\;[W_{EEG,}W_{FMRI}]\\ \;=\;arg\;max\;(A_{EEG},A_{FMRI},U_{EGG},U_{FMRI}) \end{array}$$

(4) $$\begin{array}{l} \text{max }\{H\left(Y_{EEG}\right)+H\left(Y_{FMRI}\right)+Corr{\left(A_{EEG},A_{FMRI}\right)}^{2}\}\\ \;=\;\{-E[\ln f_{y}(Y_{EEG})]\;-E[\ln f_{y}(Y_{FMRI})]\\ \;+\;\frac{Cov{\left(A_{EEGi},A_{FMRIj}\right)}^{2}}{var(A_{EEGi}).var(A_{FMRIj})}\} \end{array}$$

(5) $$\begin{array}{l} Y_{EEG}=\frac{1}{1+e^{-\left(U_{EEG}\right)}};\\ Y_{FMRI}=\frac{1}{1+e^{-\left(U_{FMRI}\right)}}; \end{array}$$

Where *f*_*y*_ (*Y*_*EEG*_) and *f*_*y*_ (*Y*_*FMRI*_) are the probability density functions of *Y*_*EEG*_ and *Y*_*FMRI*_*. Corr* is the correlation function, *Cov* is the covariance function, *E* is the expected value, *H* is the entropy function and *i* & *j* are the indices of the components.

The pICA toolbox [Calhoun, Fusion ICA Toolbox (FIT)]^1^ was used to find the parallel components as shown in Figure 1B. Please refer to Liu and Calhoun (2007) and Liu et al. (2009) for more details on the pICA algorithm.

The inputs to the parallel ICA algorithm are fMRI (Components-by-voxels) and EEG (Components-by-time points) components which are ordered such that the first fMRI component corresponds to the first EEG component and so on. In this study, we obtained 20 fMRI and EEG components from first-level ICA analysis. However, we do not know *a priori*, the correspondence between the first-level EEG and fMRI components. This information is required for them to be entered into the pICA algorithm.

**Figure 2:**
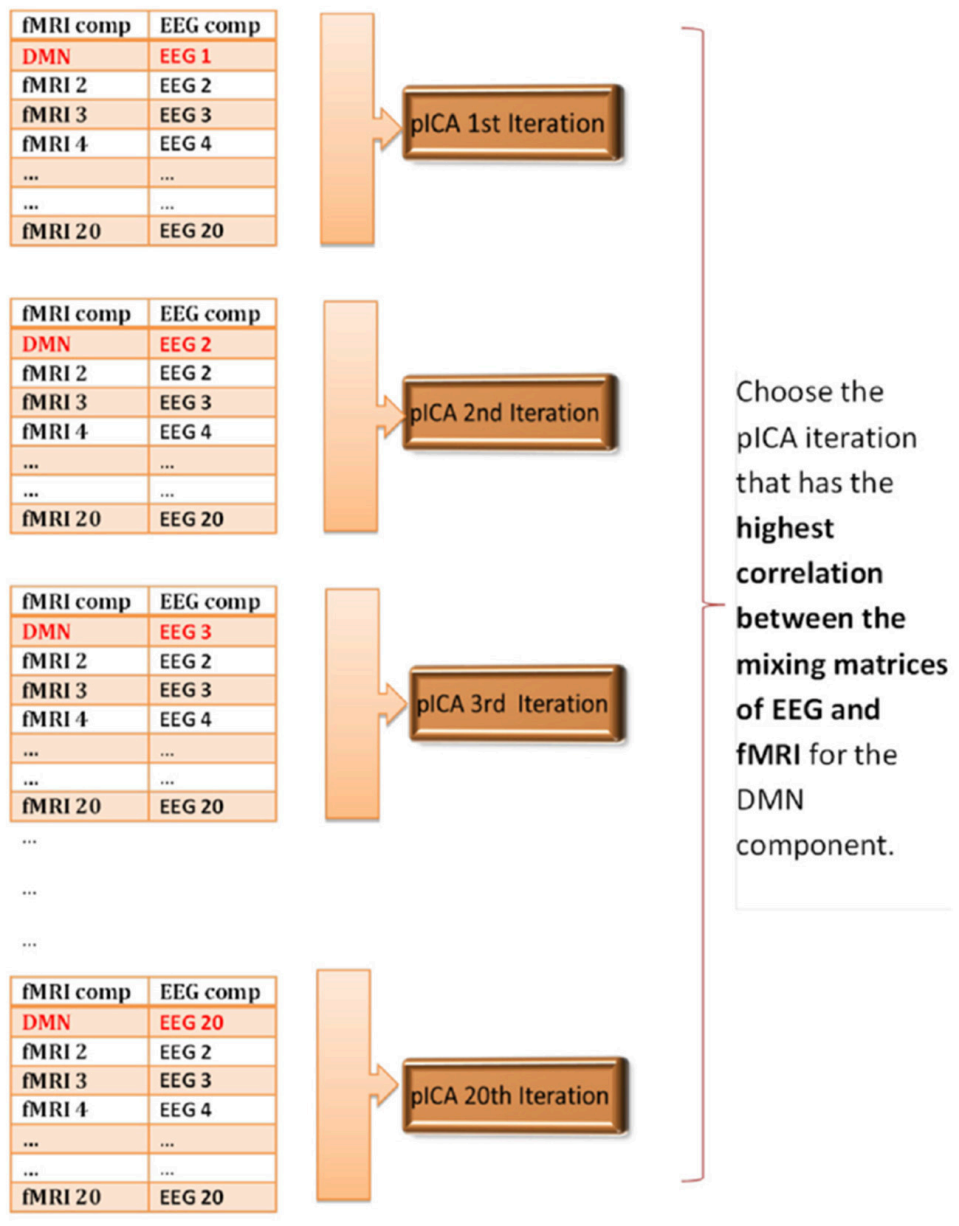
Schematic illustrating parallel ICA iterations for determining matched EEG and fMRI components for the default mode network. The correspondence between the first-level 20 EEG and 20 fMRI components is unknown, so the parallel ICA was run 20 times to see which combination of EEG component mixing matrix gives the highest correlation coefficient with the fMRI-DMN component mixing matrix.

In order to determine the correspondence between the first-level EEG and fMRI components, we made the assumption that if we applied pICA to the matched components then we should get the highest correlation between the mixing matrices of EEG and fMRI. Since we had 20 components from GIFT and 20 components from EEGIFT, so could potentially match the first-level EEG and fMRI components in 400 ways. We identified fMRI ICA components corresponding to the DMN and VN, and ran parallel ICA separately for the two networks. There are 20 ways that the DMN or VN fMRI component can be matched to the 20 first-level EEG components. So, we ran parallel ICA 20 times separately for DMN and VN to see which combination of EEG component when paired with the fMRI-DMN or fMRI-VN component gave the highest correlation between the mixing matrices. For all 20 iterations, the order of the fMRI components remained the same with the first component being DMN or VN, but the orders of EEG components were changed, with only the first EEG component, i.e., the one being paired with the fMRI DMN or fMRI VN component, being changed from component 1 to component 20 across iterations. The procedure is schematically illustrated in Figure 2 for the DMN and the procedure was similar for the VN. The idea is to keep the pairings of all components except the first one constant so that differences in the correlations between EEG and fMRI mixing matrices across iterations can be attributed to the different pairings of the first component corresponding to the DMN or VN.

## Results

**Figure 3:**
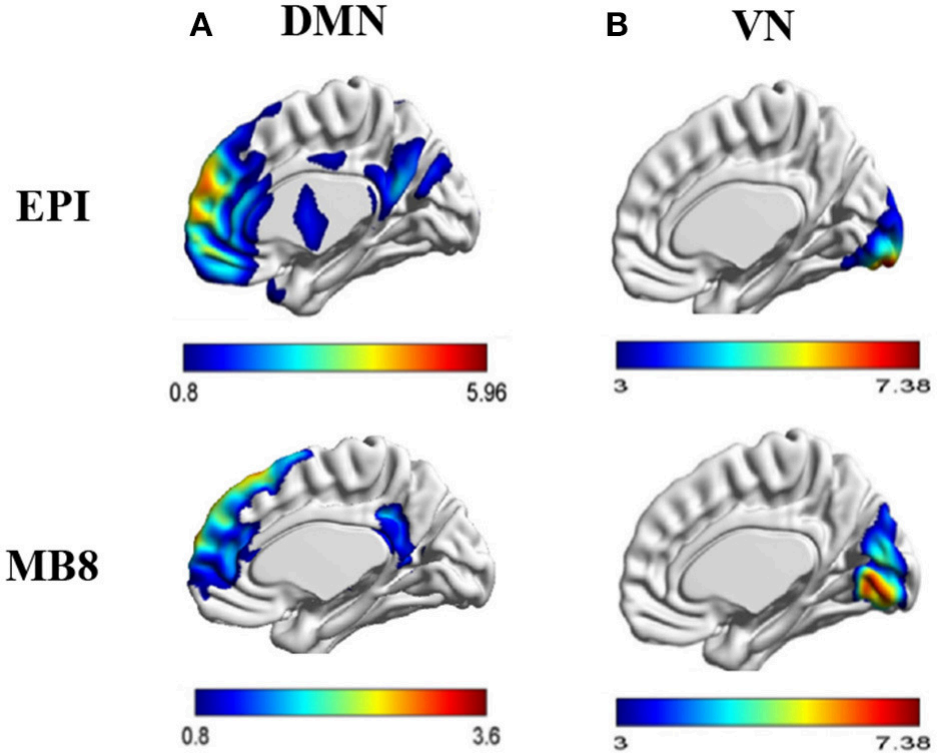
**(A)** DMN fMRI pIC map for EPI and MB8 sequences, **(B)** VN fMRI pIC map for EPI and MB8 sequences. The anatomical images used for overlay are a standard template (i.e., not derived from our data) employed in Brain Net Viewer software.

First level analysis of individual subject resting state EEG/fMRI data was carried out for data obtained from EPI (*TR* = 1,000 ms) and multiband EPI with factor 8 (MB8, *TR* = 200 ms) sequences. Using tICA and sICA, 20 temporal and 20 spatial components were obtained from EEG and fMRI, respectively, and input to a second level analysis wherein 20 parallel independent components (pICs) were derived using pICA. The EEG pIC corresponding to the fMRI pIC representing the DMN or VN was obtained by maximizing the cross correlation coefficient between their mixing matrices. The fMRI pIC maps were expressed in terms of *z*-scores with *z* > 1. Here the *z*-scores are used for descriptive purposes and have no definite statistical interpretation (McKeown et al., 1998). The resultant DMN and VN fMRI pIC map for EPI and MB8 data are shown in Figure 3.

As described previously, we paired the fMRI DMN/VN pIC with all available EEG pICs in order to determine the EEG pIC which corresponds to the fMRI DMN/VN pIC. The correlation between the mixing matrices for all the 20 iterations are summarized in Tables 1, 2 for DMN/VN obtained from EPI and MB8 data, respectively. Only one of the EEG pIC components for both EPI and MB8 data was significantly correlated (*p* < 0.05 corrected) with the fMRI DMN and VN components. This EEG pIC was assumed to represent the electrophysiological signature of the corresponding fMRI network. We wish to emphasize that it is just a co-incidence that only one EEG component was significantly correlated with respective fMRI networks (DMN and VN). It is entirely possible that multiple EEG components may be associated with resting state networks given the fact that RSNs can evolve over multiple time scales and temporal modes.

**Table 1:** The correlation between the mixing matrices for all the 20 iterations for DMN/VN obtained from EPI data.

**Iteration number**	**DMN**		**VN**	
	**Correlation coefficient**	***P*-value**	**Correlation coefficient**	***P*-value**
pICA1	−0.54	0.0135	−0.52	0.0178
pICA2	pICA did not converge		0.64	0.0024
pICA3	0.53	0.0152	0.67	0.0012
pICA4	0.55	0.0111	0.69	0.0008
pICA5	0.90	7.6 × 10^−8^	0.67	0.0011
pICA6	pICA did not converge		0.10	0.6624
pICA7	0.65	0.0018	0.57	0.0081
pICA8	0.04	0.8720	0.67	0.0012
pICA9	0.53	0.0161	0.65	0.0019
pICA10	pICA did not converge		0.50	0.0257
pICA11	−0.41	0.0713	−0.31	0.1777
pICA12	0.57	0.0088	0.66	0.0015
pICA13	0.66	0.0016	0.68	0.0011
pICA14	0.01	0.9784	0.62	0.0033
pICA15	0.18	0.4460	0.67	0.0014
pICA16	0.68	0.0069	−0.44	0.0538
pICA17	0.44	0.0498	−0.04	0.8802
pICA18	0.02	0.9260	0.17	0.4828
pICA19	−0.01	0.9544	0.64	0.0024
pICA20	0.41	0.0755	0.45	0.0479

*The 20 iterations refer to the pairing of the fMRI DMN/VN pIC with all available EEG pICs in order to determine the EEG pIC which corresponds to the fMRI DMN/VN pIC. The EEG pIC component for EPI data which was significantly correlated (p < 0.05 corrected) with the fMRI DMN and VN components is shown in bold red font*.

**Table 2:** The correlation between the mixing matrices for all the 20 iterations for DMN/VN obtained from MB8 data.

**Iteration number**	**DMN**		**VN**	
	**Correlation coefficient**	***P*-value**	**Correlation coefficient**	***P*-value**
pICA1	0.72	0.0003	0.81	1.52 × 10^−5^
pICA2	pICA did not converge		0.34	0.1408
pICA3	0.65	0.0018	−0.41	0.0723
pICA4	pICA did not converge		0.65	0.0018
pICA5	0.66	0.0012	0.66	0.0014
pICA6	0.62	0.0034	pICA did not converge	
pICA7	0.13	0.5641	0.65	0.0016
pICA8	0.27	0.2407	−0.26	0.2633
pICA9	0.10	0.6628	pICA did not converge	
pICA10	pICA did not converge		0.55	0.0108
pICA11	0.65	0.0018	0.67	0.0012
pICA12	−0.17	0.4753	0.66	0.0013
pICA13	0.59	0.0055	0.64	0.0021
pICA14	0.64	0.0023	0.61	0.0038
pICA15	0.66	0.0015	0.68	0.0009
pICA16	pICA did not converge		0.03	0.894
pICA17	0.54	0.0130	0.48	0.0293
pICA18	0.66	0.0014	0.63	0.0025
pICA19	0.65	0.0016	0.63	0.0025
pICA20	−0.61	0.0040	−0.35	0.1241

*The 20 iterations refer to the pairing of the fMRI DMN/VN pIC with all available EEG pICs in order to determine the EEG pIC which corresponds to the fMRI DMN/VN pIC. The EEG pIC component for EPI data which was significantly correlated (p < 0.05 corrected) with the fMRI DMN and VN components is shown in bold red font*.

It was shown in previous studies (Liu and Calhoun, 2007; Liu et al., 2009) that the parallel ICA algorithm is robust and shows improved performance compared to regular ICA (Infomax). In order to investigate whether the pICA algorithm provided any advantage over simply correlating the mixing matrices found from the application of regular spatial and temporal ICA for our data, we took the mixing matrices corresponding to fMRI DMN/VN component (from GIFT) and the EEG mixing matrices (for 20 components- from EEGIFT) and found the correlation between those mixing matrices. Table 3 shows the maximum correlation values between the mixing matrices of two modalities found by applying parallel ICA (2nd level analysis) and regular ICA (1st level analysis). It can be seen that the correlation values found for regular ICA are lower compared to the correlation values found after using parallel ICA.

**Table 3:** Correlation and its corresponding *p*-value between mixing matrices of EEG and fMRI pICs for EPI and MB8 data using both Regular ICA and Parallel ICA.

	**Regular ICA**		**Parallel ICA**	
**Condition**	**Maximum correlation**	***p*-value**	**Maximum correlation**	***p*-value**
**A. DMN**				
EPI	0.43	0.057	0.90	7.6 × 10^−8^
MB8	0.40	0.08	0.81	1.5 × 10^−5^
**B. VN**				
EPI	0.36	0.15	0.69	8.0 × 10^−4^
MB8	0.39	0.06	0.72	3.0 × 10^−4^

*The results are shown for both the DMN **(A)** and VN **(B)***.

**Figure 4:**
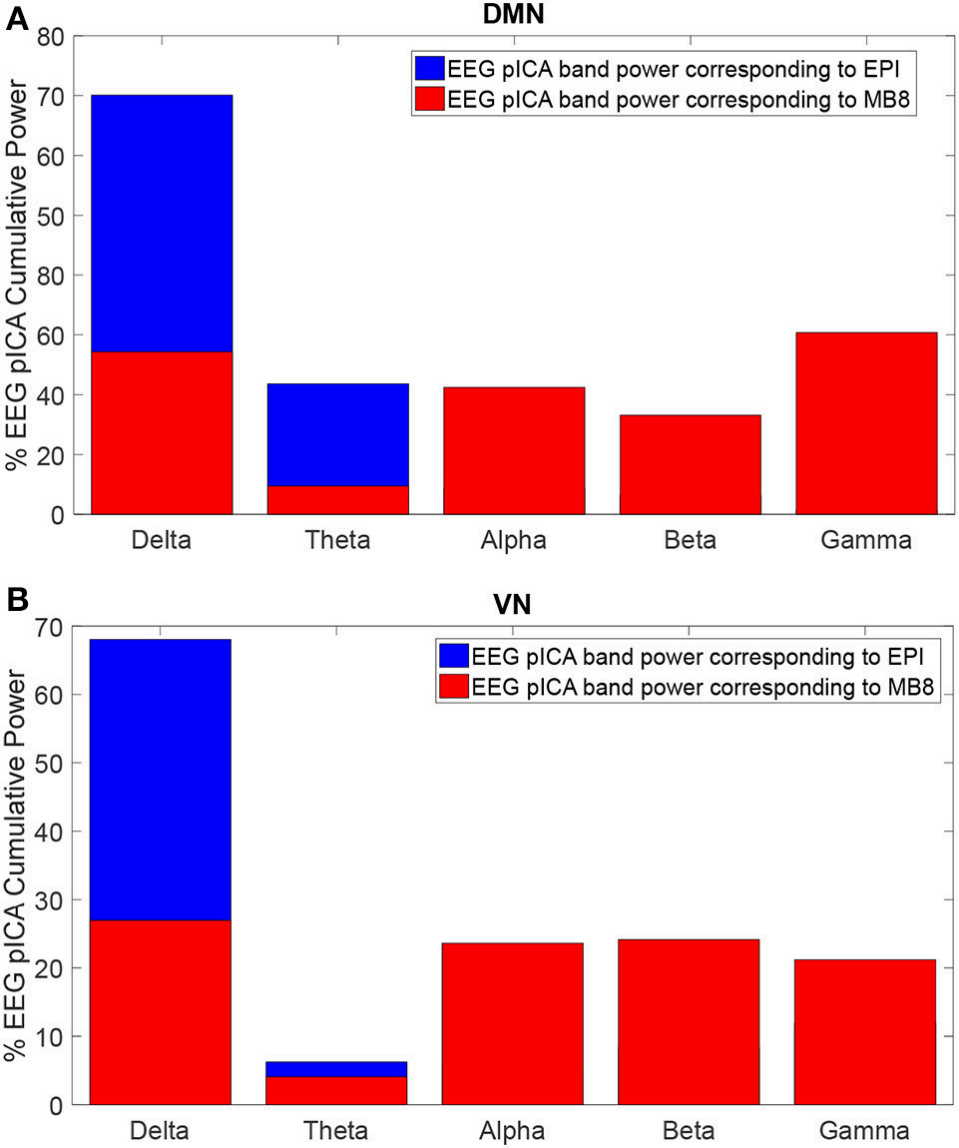
Percentage cumulative power in different frequency bands of the EEG pIC corresponding to the DMN **(A)** and VN **(B)** for EPI (blue) and MB8 (red) for eyes open conditions. The power of the EEG pIC was greater in lower frequency bands for regular EPI data whereas for MB8 data, the power was distributed across frequency bands and had greater power at higher frequency when compared to EPI data.

The EEG component corresponding to DMN or VN fMRI component for both EPI and MB8 condition was considered and its power spectral density was calculated. The percentage of cumulative power spectral density vs. frequency was plotted for the linked DMN EEG pIC for EPI (Figure 4A, blue) and MB8 (Figure 4A, red), as well as for the VN EEG pIC for EPI (Figure 4B, blue) and MB8 (Figure 4B, red). The frequency bands considered here are delta band from 0 to 4 Hz, theta band 5 to 8 Hz, alpha band from 9 to 12 Hz, beta band from 13 to 30 hz, and gamma band from 31 to 60 Hz. From both DMN and VN, the percentage of cumulative power of the corresponding EEG pIC was relatively larger in lower frequency bands for regular EPI data (Figures 4A,B, blue), whereas for the MB-EPI data (Figures 4A,B, red) the relative power was more distributed across frequency bands with more power at higher frequencies (beta, gamma) when compared with EPI data. Table 4 shows the percent cumulative EEG power values in all frequency bands for EPI and MB8 data. Note that the power at lower frequency bands such as delta, theta and alpha was higher for EPI data compared to MB8 data whereas the power at higher frequency bands such as beta and gamma was lower for EPI data compared to MB8 data.

**Table 4:** The percentage of cumulative EEG power obtained from corresponding pICs in all relevant EEG frequency bands for EPI and MB8 data.

**MRI Sequence**	**Delta (%)**	**Theta (%)**	**Alpha (%)**	**Beta (%)**	**Gamma (%)**
**A. DMN**					
EPI	70.2	21.9	4.3	3.2	0.5
MB8	36.3	6.3	28.2	22.0	7.2
**B. VN**					
EPI	68.1	6.2	5.5	8.3	11.9
MB8	28.9	4.1	23.7	24.2	21.2

*The results are shown for both the DMN **(A)** and VN **(B)***.

**Figure 5:**
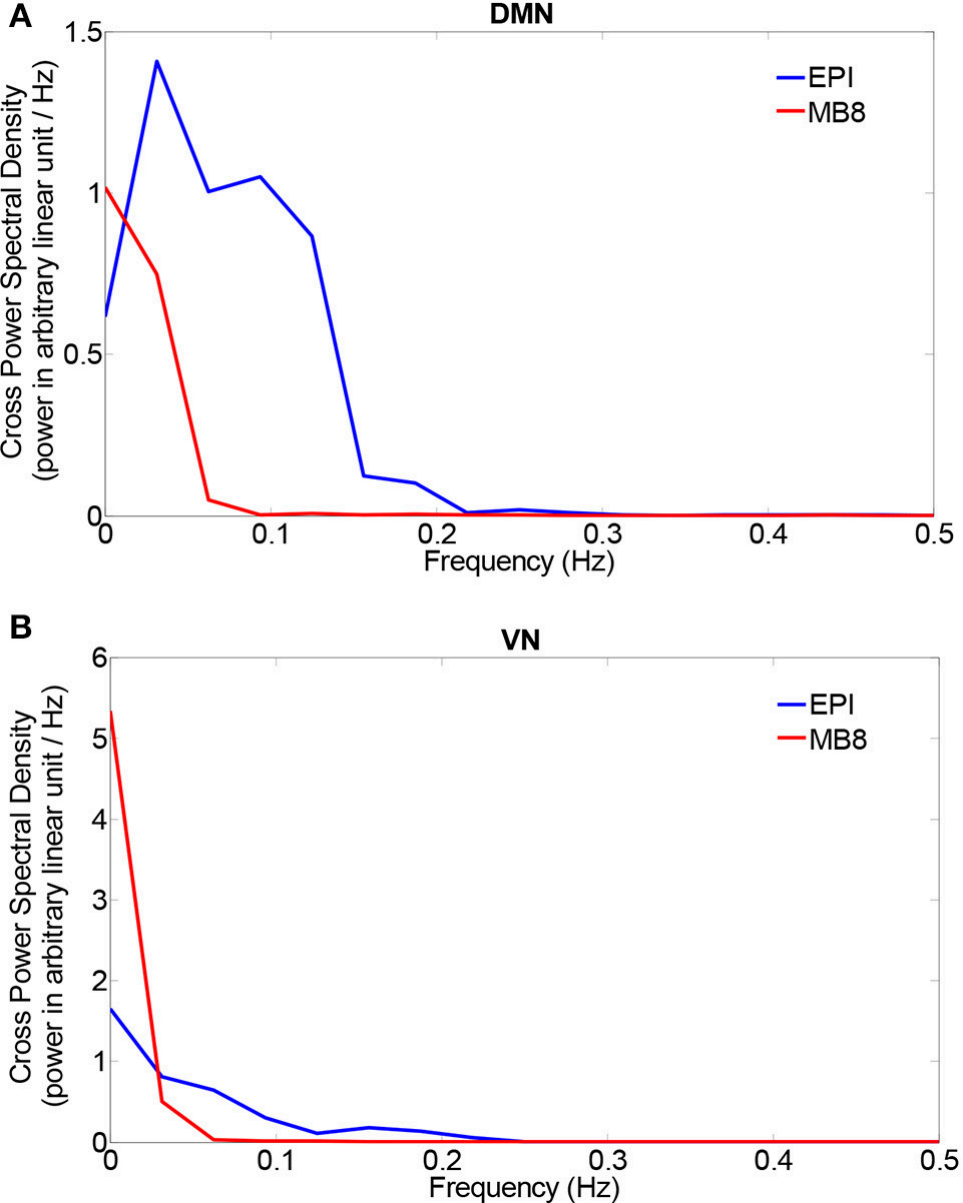
Cross power spectral density between mean fMRI time series and a HRF-convolved and down-sampled version of the EEG pIC time series from two networks [DMN **(A)** and VN **(B)**].

The chosen EEG component from pICA was convolved with a standard canonical HRF and downsampled (to match fMRI sampling frequency). We then separately calculated the cross power spectral density (CPSD) between the mean fMRI time series extracted from the DMN/VN and the corresponding convolved, downsampled EEG pIC time series using in-built MATLAB code based on Welch's method. The CPSD shown in Figure 5 shows predominant low frequency (range of [0.008, 0.1] Hz) cross-spectral power, indicating that when EEG pICs corresponding to DMN and VN are convolved with the HRF and downsampled, they are correlated with corresponding fMRI time series in the low frequency bands ranging from 0.008 to 0.1 Hz. Subsequently, we calculated time-domain correlation between mean fMRI time series obtained from DMN/VN and corresponding HRF-convolved and downsampled EEG pIC time series. The results, shown in Table 5 indicates that they were significantly correlated. Further, similar analysis using time series obtained from other EEG pICs did not reveal a significant correlation with fMRI time series obtained from DMN/VN. This suggests that the chosen EEG pIC time series are more likely to be associated with corresponding fMRI networks (DMN and VN).

**Table 5:** The correlation coefficient (and corresponding *p*-values) between the mean fMRI time series and the corresponding HRF-convolved, downsampled EEG pIC time series for DMN **(A)** and VN **(B)**.

**Condition**	**Correlation**	***p*-value**
**A. DMN**		
EPI	0.23	2.40 × 10^−11^
MB8	0.20	2.26 × 10^−19^
**B. VN**		
EPI	0.11	3.10 × 10^−3^
MB8	0.16	3.75 × 10^−18^

## Discussion

The parallel ICA method for combining fMRI and EEG enabled us to evaluate relationships between resting state network maps obtained from the former and electrical time courses obtained from the latter. These results demonstrate that: (i) electrical substrates of RSNs (DMN and VN) consist of both low and high frequency fluctuations, (ii) faster fMRI sampling is required to reveal RSNs' high frequency electrical substrates, (iii) the association of RSNs with fast electrical dynamics proves that its neural origin is relevant to typically occurring fast mental processes, and (iv) high frequency electrical substrates of RSNs may support the notion that resting state fluctuations reveal scale-free fractal properties. Below, we expand on these themes.

Over past several years, researchers have tried to show the link between the EEG frequency bands and BOLD-fMRI based RSNs and some have claimed that the EEG power envelope at specific frequency bands correlates with RSNs (Goldman et al., 2002; Laufs et al., 2003; Moosmann et al., 2003). Others have argued and showed that EEG is made up of a wide frequency spectrum (Varela et al., 2001; Buzsaki and Draguhn, 2004), and thus the correlation between neuronal electrical activity only at specific frequencies to the BOLD RSNs cannot be true. This notion was supported by Mantini et al. who demonstrated electrical correlates of different EEG spectral band envelopes with the fMRI RSNs (Mantini et al., 2007) thus proving that fMRI RSNs have unique correlation patterns across frequency bands. Since these studies have convolved power time courses and/or their envelopes of the bands of interest with a hemodynamic response function and subsequently downsampled it, they have ignored truly high frequency dynamics of the electrical signal. Therefore, they cannot really investigate whether high frequency electrical substrates of RSNs exist or not. Consequently, it is unclear whether RSNs can be associated with typical neuronal processes with periods <100 ms. On the other hand, a recent study used empirical mode decomposition to show that resting state fMRI fluctuations are in fact broad band processes though their energy is concentrated in low frequencies (Niazy et al., 2011). Another recent study on fMRI RSNs investigated relatively higher frequency signal fluctuations (>0.25 Hz) using data acquired with a low TR of 354 ms and showed using temporal ICA that RSNs including the DMN exist at relatively higher frequencies than previously thought (Boubela et al., 2013). Unlike our study, these two recent studies did not look at electrical substrates of DMN or VN, but they do suggest that even by just looking at fMRI data alone, one can come to the conclusion that fluctuations with a frequency higher than 0.1 Hz are not to be ignored. In fact, previous studies have shown that the 0.1–0.25 Hz range is clinically significant (Garrity et al., 2007; Calhoun et al., 2008; Allen et al., 2011). In our study, since we did not downsample the EEG or consider its power envelope, our results with fMRI data acquired with faster sampling indicate that both low and high frequency electrical substrates exist for the DMN. These results demonstrate that faster sampling is required to find high frequency electrical substrates of RSNs. It is thus important for resting state fMRI studies to use as small a TR as possible.

Previous studies indicate that many sensory neuronal processes occur during 50–200 ms post stimulus (Di Russo et al., 2002; Sadeh et al., 2008), while neuronal processes involving cognition happen during the 100–600 ms interval (Kutas et al., 1977; Gouvea et al., 2010). It is thus well-known that neuronal dynamics occurs at a sub-second time scale. On the other hand, typical studies involving BOLD RSNs perform band pass filtering of fMRI time series in the frequency range of 0.01–0.1 Hz. This is done because fMRI has a dominant low frequency spectrum due to hemodynamic smoothing and filtering out frequencies higher than 0.1 Hz can get rid of some sources of noise. This indicates that the period of the fastest variation in the signal is 10 s. This is at odds with the typical fast neuronal dynamics; however, given the sensitivity of BOLD RSNs, especially the DMN and VN, to brain pathology (Buckner et al., 2008; Mevel et al., 2010; Bonnelle et al., 2011; Shin et al., 2011; Mingoia et al., 2012; Zhou et al., 2012), there is reason to believe that the RSNs have a neural basis. This apparent contradiction can be reconciled by our findings that with faster sampling and pICA, truly high frequency electrical substrates of RSNs can be revealed in the beta (13–30 Hz, periods: 34–77 ms) and gamma bands (30–60 Hz, periods 17–34 ms). Note that unlike previous studies (Mantini et al., 2007; Britz et al., 2010; Musso et al., 2010) which showed that the downsampled versions of the power envelopes of these high frequency bands, which are in effect lower frequency fluctuations, correlated with fMRI time series fluctuations in the RSNs, we have demonstrated, without any downsampling or using power envelopes, that the electrical substrates of the RSNs exist in high frequencies. In our study, the link between electrical activity in EEG and fMRI spatial patterns is made based on similar inter-subject variability in these modalities captured by pICA. Our findings may support the notion that fluctuations of brain activity at rest are scale-free, as discussed below.

Studies have shown that EEG microstates are potential electrophysiological substrates of fMRI RSNs (Britz et al., 2010; Musso et al., 2010). Microstates are scalp topological configurations which remain quasi-stable for 80–100 ms. Previous studies (Britz et al., 2010; Musso et al., 2010) showed that microstate time series (a quantized signal obtained by the dynamic state transitions between microstates) when convolved with a hemodynamic response function and downsampled to the fMRI resolution, correlated with BOLD time series derived from RSNs. While Britz et al. (Musso et al., 2010) did not find a microstate time series corresponding to the DMN, Musso et al. (Britz et al., 2010) and Yuan et al. (2012) did. Even though these studies used convolved and downsampled versions of microstate time series, they speculated that since microstates themselves represent fast neuronal dynamics, the RSNs such as the DMN may have electrical substrates in high frequencies. Consequently, they postulated that brain dynamics at rest may be scale-free such that their correlational structure will be visible at any time scale. Subsequently, it was shown that EEG microstates were indeed scale-free (Van de Ville et al., 2010). The above evidence, taken together with our results on both low and high frequency electrical substrates of RSNs, support the notion that resting state BOLD fluctuations may have scale-free fractal properties.

One limitation of this study is that we had to use different TEs, flip angles and slightly different voxel sizes for regular EPI and MB8 acquisitions because we wanted to optimize the sequence for minimum TR and whole brain coverage. However, given that ICA is pretty robust to differences in SNR, we believe that our results would be replicated even if data from different sequences were acquired using identical parameters. Further, we have shown that pICA confers benefits in extracting temporal information regarding spatial RSNs from EEG/fMRI data; however, others have shown that pICA also improves spatial specificity. For example, Hunyadi et al demonstrated a circumstance in which pICA of EEG/fMRI data pinpointed an epileptic foci whereas the conventional GLM-based EEG-correlated fMRI analysis only identified a broad epileptogenic network (Hunyadi et al., 2015). We have not investigated the potential benefits that pICA might offer in terms of improved spatial specificity.

In summary, we used an integrated framework involving simultaneous EEG/fMRI acquisition, fast fMRI sampling (*TR* = 200 ms) using multiband EPI, and EEG/fMRI fusion using pICA, to test the hypothesis that resting state fluctuations in fMRI may be associated with not only low frequency, but also high frequency electrical dynamics. The salient feature of our approach is that we did not convolve EEG features with a hemodynamic response function or down sample EEG to fMRI temporal resolution. This allowed us to make inferences about truly fast electrical dynamics with periods <100 ms related to resting state fMRI fluctuations in RSNs such as DMN and VN.

## Author contributions

GD designed the experiment. SK acquired data and performed data analysis. YW performed data analysis. VC contributed analysis tools, all authors wrote the paper.

### Conflict of interest statement

The authors declare that the research was conducted in the absence of any commercial or financial relationships that could be construed as a potential conflict of interest.

## Footnotes

^1^http://mialab.mrn.org/software/fit/
